# Association between nutritional status, physical fitness and executive functions in preadolescents: A person-centered approach

**DOI:** 10.3389/fped.2022.966510

**Published:** 2022-10-17

**Authors:** Yuxin Zhu, Fenghua Sun, Sisi Tao, Simon B. Cooper, Tian-Yu Gao

**Affiliations:** ^1^ Syns Institute of Educational Research, Hong Kong, Hong Kong SAR, China; ^2^ Department of Health and Physical Education, The Education University of Hong Kong, Hong Kong, Hong Kong SAR, China; ^3^ Centre for Information Technology in Education, Faculty of Education, The University of Hong Kong, Hong Kong, Hong Kong SAR, China; ^4^ Exercise / Health Research Group, Sport, Health and Performance Enhancement (SHAPE) Research Centre, Department of Sport Science, Nottingham Trent University, Nottingham, United Kingdom; ^5^ School of Physical Education, Jinan University, Guangzhou, China

**Keywords:** body mass index, exericse, latent profile analysis, inhibition, working memory, attention, cognition

## Abstract

**Objective:**

In the current study, a person-centered approach was adopted to investigate the relationship between nutritional status and physical fitness profiles and executive functions (EF) in preadolescents.

**Methods:**

Participants (*M*_age _= 10.8 years; Male = 50.8%) were recruited from two primary schools in Hong Kong. Nutritional status [body mass index (BMI)], physical fitness including cardiorespiratory fitness (CRF, predicted VO_2max_, multi-stage fitness test) and speed-agility (20-m sprint) were measured on school days. EF performance was measured using the Flanker task (inhibition) and the Sternberg task (working memory).

**Results:**

Data from 120 preadolescents were considered valid. Three distinct profiles were identified by a person-centered approach. Profile 1 was featured by high BMI (21.61 ± 3.38 kg/m^2^), poor VO_2max_ (33.29 ± 23.96 ml/kg/min), and slow 20-m sprint (4.51 ± 0.13 s). Profile 2 was featured by low BMI (15.99 ± 3.38), fair VO_2max_ (44.98 ± 23.96) and fast 20-m sprint (3.97 ± 0.13). Profile 3 was featured by low BMI (15.63 ± 3.38), poor VO_2max_ (32.37 ± 23.96), and slow 20-m sprint (4.48 ± 0.13). Wald chi-square test revealed preadolescents in profile 1 and profile 2 performed better than profile 3 in accuracy of Flanker task (1 vs. 3: *χ*^2^ = 12.23, *P* < 0.001; 2 vs. 3: *χ*^2^ = 10.86, *P* = 0.001). That is, for normal weight preadolescents with poor CRF and speed-agility, those with superior nutritional status performed better in inhibition. For normal weight preadolescents with poor nutritional status, those with superior CRF and speed-agility had better inhibitory capacity.

**Conclusion:**

Compared to the commonly used variable-centered approach, this person-centered approach is a valuable addition that expands the understanding of the association between nutritional status, physical fitness and EF in preadolescents. Results are discussed with regards to maximizing health behaviors and implications for educational policy.

## Introduction

Executive function (EF) refers to high-level, self-regulatory neurocognitive processes that help monitor and control thoughts and goal-directed behavior (1). Components of EF include inhibitory control, interference control, working memory, and cognitive flexibility (1). EF is critical for children and adolescents' academic performance and serves as a cornerstone for social behaviors that are exhibited across the lifespan (1). For children and adolescents, nutritional status and physical fitness have been proposed to be closely related to EF performance (2, 3). Understanding the correlation between the integration of nutrition status and physical fitness on domain-specific EF performance may provide insights into the potential of dietary behaviors and physical activity-based interventions to improve EF performance in children and adolescents.

To date, most studies examining the relationship between nutritional status and physical fitness on EF performance in children and adolescents have used a variable-centered approach (i.e., describing and providing information on the strength of the associations between variables, such as regression) and yielded inconsistent findings. Body mass index (BMI), the most commonly used and simplest indicator of nutritional status, was generally reported to be reversely associated with EF performance (i.e., higher BMI is associated with poorer EF performance, especially for obese children) (4, 5). However, cross-sectional studies found that in school-aged children, there was no significant association between BMI and attention (6, 7), working memory (6), and cognitive processing (8). The components of cardiorespiratory fitness (CRF), muscle strength, and motor capacity are key indices of physical fitness that are reported to be associated with EF performance in children and adolescents (3, 9). Esteban-Cornejo et al. (2017) observed that CRF and motor capacity but not muscular strength was associated with greater gray matter volume in different cortical regions (i.e., frontal, temporal and calcarine cortices) of the brain (10), which in turn, affect the children' EF (11). Controversially, one experimental study adopting advanced functional magnetic resonance imaging (fMRI) found that cardiorespiratory fitness (CRF) was not predicting inhibition control (measured by Flanker task) in preadolescents (12). Ruiz et al. (2010) reported that physical activity during leisure time positively influenced EF performance, but the beneficial effect was independent of CRF and BMI (13). The discrepancies suggest that the variable-centered approach may not be able to reveal the complex association between nutritional status and physical fitness on EF performance, as this approach only considers associations that are identified across a sample to summarize the population with a single set of parameters (14).

Notably, the correlation between nutritional status and physical fitness underscores the need to view them as a holistic concept that influences EF in children and adolescents (15, 16). In recent years, a person-centered approach—latent profile analysis—has been developed and is attracting growing interest in the sports field. Latent profile analysis is a categorical latent variable modeling approach that focuses on identifying latent subpopulations within a population based on a certain set of variables (17). Compared to the traditional variable-centered approach, this person-centered approach identifies individuals who share similar patterns of variables in the same profile and compares them to other profiles, both in terms of how the variables are combined to form the profiles and how these combinations relate to other variables (e.g., demographic characteristics) (17). The profiles may provide additional insight into preadolescents' alignments of their nutritional status and physical fitness, as well as their collective association with EF. Specifically, this approach allows investigation of whether nutritional status and physical fitness are aligned in most children (e.g., good nutritional status, CRF, and speed-agility) or if some children have divergent levels of these indices (e.g., good nutritional status, poor CRF and speed-agility). It may also contribute to a better understanding of EF in preadolescents by examining the combined importance of nutritional status and physical fitness in relation to EF performance and determining whether children with different profiles of nutritional status and physical fitness differ in their EF performance. Studies using both a variable-centered and a person-centered approach have yielded the consistent (18) or conflicting (19) results on the relationships between a particular set of key variables, suggesting that the use of latent profile analysis may provide additional information on relationships from a more nuanced person-centered perspective.

Previous empirical studies have adopted latent profile analysis in the sports field. For example, Berlin et al. (2017) found that students' physical activity, sedentary behavior and nutritional choices profiles predicted BMI and psychosocial functioning (20). Cheung and Li (2019) identified three academic burnout profiles and reported that the “well-functioning group” reported significantly higher levels of physical activity and mental toughness than the other two groups (21). To date, however, no study has used latent profile analysis to investigate the relations between nutritional status and physical fitness profiles on EF performance. Understanding the profiles of preadolescents' nutritional status and physical fitness could help identify preadolescents with similar patterns of these indices and examine the relationships between the profiles and EF performance from an individual perspective. The research questions of this study were: (1) what are the latent profiles of nutritional status and physical fitness of preadolescents? (2) what are the relations between the latent profiles of nutritional status and physical fitness and EF performance of preadolescents?

## Materials and method

### Participants

A total of 184 right-handed children from two elementary schools in Hong Kong were recruited by convenience sampling. Of them, 120 children (male = 50.8%) who completed anthropometric measures, physical fitness and EF tests are considered valid data. The remaining 64 participants were excluded from the analysis because they were absent from one or more assessments. The mean age was 10.8 (±0.5) years with a chronological range of 10–12 years (see Table 1, Participants' characteristics). Children with color blindness, sensory deficits, special needs, or a condition in which movement is contraindicated and could interfere with testing were excluded. Informed consent was obtained from the children's parents/guardians before the study. The University Ethics Committee approved the protocol in accordance with the Declaration of Helsinki (No. 2017-2018-0404).

**Table 1:** Participants' characteristics (*N* = 120).

Preadolescents	Mean	SD
Age (years)	10.8	0.5
Height (cm)	144	8
Weight (kg)	36.6	9.1
BMI (kg/m^2^)	17.25	3.13
VO_2max_ (ml/kg/min)	37.22	7.71
20-m sprint (sec)	4.3	0.44

BMI, Body mass index.

### Procedure

The study was a cross-sectional study. Prior to the tests, a screening survey was conducted to exclude ineligible individuals. Data were collected in each school for two consecutive weeks within one semester. Participants’ weight and height were collected barefoot and lightly clothed in the school. The EF tests were conducted using a laptop in a quiet classroom at the school at a constant temperature of 22 °C. Participants were instructed to practice the test battery twice before the formal test to familiarize themselves with the tasks and avoid the learning effect. The order of the tasks was consistent during the assessment, i.e., the Flanker task (FT) was completed first, followed by the Sternberg task (SBT), and there was a 1-minute break between the two tests. The CRF and 20-m sprint were assessed in PE class by a trained research assistant with the assistance of the school PE teacher. After a 10-min warm-up (400-m jog and stretch), the 20-m sprint was assessed first. After a break of several minutes, participants were instructed to complete the 15-m version of the multi-stage fitness test on an outdoor playground to determine maximal oxygen consumption (VO_2max_) for CRF. Anthropometric measurement (weight and height) and EF tests were measured in the first phase, followed by the second phase of the CRF test and 20-m sprint. The specific time for conducting the measurements depends on the availability of the children and teachers in the school.

### Measurements

Nutritional status was assessed using anthropometric measurements, expressed as BMI in kg/m^2^ (22, 23). The categories of nutritional status referred to the World Health Organization (WHO) recommendation for children aged 5–19 years, with overweight is defined as a BMI-for-age value greater than 1 SD, obesity as a BMI-for-age value greater than 2 SD and underweight as BMI-for-age less than 2 SD of the mean (23, 24). The BMI value of our sample was compared to a large sample study of Chinese urban primary children expressed in the form of a percentile grid (Cut-off value for overweight >21.61, obesity >24.87 and underweight <11.83 kg/m^2^) (25).

Cardiorespiratory fitness was measured using a 15-m version of the multi-stage fitness test (i.e., VO_2max_) (26). The test required participants to run between two labeled lines 15-m apart. The running speed started at 8.0 km/h, then increased to 9.0 km/h, and increased 0.5 km/h for every minute. Participants were required to shuttle run following the audio instruction to the point of volitional exhaustion or until they could no longer keep pace with the audio signal. The Ramsbottom equation was used to predict the VO_2max_ from the performance of the multi-stage fitness test (26).

Speed-agility was measured by 20-m sprint. The sprint began after a signal was issued by PE teacher with an upright posture at the start line and recorded the performance at the stop line by a research assistant *via* a stopwatch. Participants were instructed to sprint twice (sprint and walk back for a second sprint). The best performance was recorded for further analysis.

Two widely used tasks in a computerized battery were adopted to assess EF performance, which have been described and successfully applied in several studies (27–32). Each test was preceded by six practice stimuli to re-familiarize the participant with the task and negate any potential learning effects. The Flanker Task assesses selective attention and the inhibitory control domain of EF (33). In this task, the target is located in the center and flanked by non-target stimuli. Participants were requested to press the left or right arrow key corresponding to the direction of the target. There are two types of non-target stimuli in the FT, congruent and incongruent. In the congruent condition, the direction of the non-target stimuli is the same as that of the target, while in the incongruent condition, the direction of the non-target stimuli is the opposite direction of the target. Each target and its corresponding non-target stimuli appear for 3 s, and there is a one-second pause between each target. The task takes an average of 3 min to complete. The task has a total of 60 trials. The response time of correct responses and the proportion of correct responses made were recorded for analysis.

The Sternberg task assesses how individuals store and retrieve random information from short-term memory, and measures the working memory domain of EF (34). The task consists of three ascending levels with a total of 80 trials. Participants were instructed to remember a random number or a series of letters with a random sequence, starting with the one-item level (easy mode, to remember one single number) and followed by the three- and five-item levels (hard mode, to remember five random letters). Once the letter disappeared from the screen, a single number/letter was presented in the center of the screen, and participants were required to recall if this number/letter was present in the previous letter set or not as quickly as possible. If the number/letter had previously been presented, participants should press the right arrow. If the number/letter had not previously been presented, the left arrow key should be pressed. The task takes approximately 5 min to complete. The response time of correct responses and the proportion of correct responses made were recorded for analysis.

### Statistical analysis

Statistical analyses were conducted in Mplus Version 8.1. BMI, VO_2max_, and 20-m sprint were subjected to a robust maximum likelihood estimation of latent profile analysis. In the analysis, 1,000 random starting values were used to ensure the validity of each class solution. The number of latent classes (groups) was determined as follows. Starting with a single latent class, additional classes were added in sequence, until a model was found that met optimal selection criteria. In the present study, the optimal statistical number of classes was determined using the Bayesian Information Criterion (BIC), the sample-size Adjusted BIC (ABIC), the Lo-Mendell-Rubin likelihood ratio test (LRT), and the Adjusted LRT (ALRT). Lower BIC and ABIC values indicate a better model. The LRT and the ALRT test a model with K classes vs. a model with K-1 classes. A significant *P*-value indicates that the model with K classes is better than the model with K-1 classes. A non-significant *P*-value indicates that the model with K classes does not improve the model with K-1 classes. Although entropy is generally not used to determine the model with the optimal number of classes, it is useful as it summarizes classification accuracy (whether individuals are classified neatly into one and only one category). Entropy varies from 0 to 1, with values closer to 1 indicating fewer classification errors. The Entropy above 0.76 is associated with at least 90% correct assignment for 3 latent classes, and above 0.84 for 5 latent classes, respectively (35). The final model was chosen based on both statistical results and theoretical implications.

The relations between profiles and constructs related to EF (i.e., response times and accuracy on the FT and SBT) were examined by Wald chi-square tests [i.e., Bolck, Croon, and Hagenaars (BCH) method]. The BCH procedure is the robust and recommended method for examining relationships between classes and continuous variables (36).

## Results

### Latent profiles

To identify the optimum number of BMI, VO_2max_, and 20-m sprint profiles, we computed models with 2–5 profiles. Table 2 provides BIC, ABIC, LRT, ALRT and entropy models. Both the BIC and ABIC decreased sequentially from the 2- to 3- to 4-profiles. The BIC value for the 4-profiles model was slightly lower than that of the 3-profiles model (ΔBIC = −6.2), and the ABIC value for the 4-profiles was lower than the 3-profiles model (ΔABIC = −18.85). The BIC was higher in the 5-profiles model than the 4-profiles model (ΔBIC = 7.85), and the ABIC was lower in the 5-class model than the 4-class model (ΔABIC = −4.79). The LRT and ALRT values for the 2-profiles LPA solution were significant at *P* < 0.001, and for the 3-, 4- and 5-profiles LPA solutions were not significant. Collectively, these findings do not support the 5-profiles model, and it is not necessary to test models with more profiles. The overall classification accuracy (Entropy) was 0.73 for the 2-profile model, 0.78 for the 3-profiles model, 0.82 for the 4-profile model and 0.8 for the 5-profile model.

**Table 2:** Fit statistics of the latent profile analysis models.

Model	BIC	Adjusted BIC	LRT *P*-value	Adjusted LRT *P*-value	Entropy
2-class	1578.54	1546.92	0.00	0.00	0.73
3-class	1570.99	1526.73	0.19	0.20	0.78
4-class	1564.79	1507.88	0.14	0.15	0.82
5-class	1572.64	1503.09	0.67	0.68	0.80

BIC, Bayesian information criterion; LRT, Lo-Mendel Rubin likelihood ratio test.

**Figure 1:**
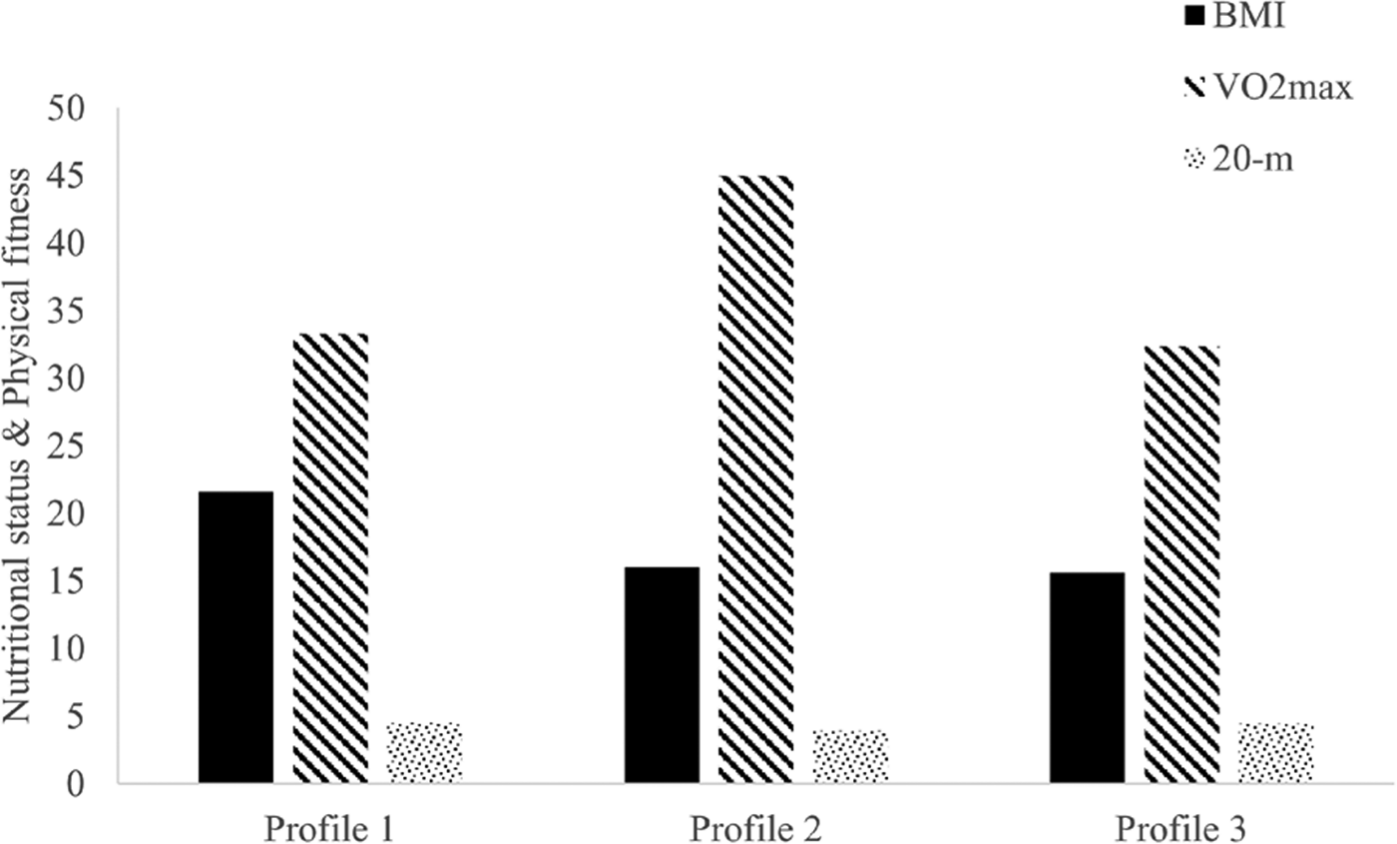
Characteristic of classified three profiles. Note: BMI, Body mass index, values are within the Normal weight range for three profiles. Profile 1—high BMI, poor VO_2max_, and slow 20-m sprint; Profile 2—low BMI, fair VO_2max_, and fast 20-m sprint; Profile 3—low BMI, poor VO_2max_, and slow 20-m sprint.

Although the LRT and ALRT results support the 2-profiles model, the improvement of the 3-profiles model over the 2-profiles model was observed in BIC, ABIC and Entropy. The 4-profiles model also increased in BIC, ABIC and Entropy compared to the 3-profiles model. For the 3-profiles model, the percentage of individuals correctly classified were 94% for profile 1, 91% for profile 2, and 87% for profile 3. For the 4-class model, the percentage of individuals correctly classified were 88.7% for profile 1, 85.6% for profile 2, 95% for profile 3, and 87.9% for profile 4. These findings indicate greater parsimony for the 3-profiles model than the 4-profiles model. Thus, the 3-profiles model was applied in the current study. Profile 1, 2, and 3 consisted of 23.33% (*N* = 28), 35.83% (*N* = 43), and 40.83% (*N* = 49) of the sample, respectively. The gender composition was balanced among three classified profiles (*χ*^2^ = 0.06). Profile 1 was featured by high BMI (21.61 ± 3.38 kg/m^2^), poor VO_2max_ (33.29 ± 23.96 ml/kg/min), and slow 20-m sprint (4.51 ± 0.13 s). Profile 2 was characterized by low BMI (15.99 ± 3.38 kg/m^2^), good VO_2max_ (44.98 ± 23.96 ml/kg/min) and fast 20-m sprint (3.97 ± 0.13 s). Profile 3 was featured by low BMI (15.63 ± 3.38 kg/m^2^), poor VO_2max_ (32.37 ± 23.96 ml/kg/min), and slow 20-m sprint (4.48 ± 0.13 s) (See Figure 1 and Table 3).

**Table 3:** Characteristic of classified three profiles.

	BMI (kg/m^2^)	VO_2max_ (ml/kg/min)	20-m sprint (sec)
Profile 1	21.61 (0.52)	33.29 (1.4)	4.51 (0.12)
Profile 2	15.99 (0.45)	44.98 (1.26)	3.97 (0.05)
Profile 3	15.63 (0.35)	32.37 (1.02)	4.48 (0.06)

Data presented as mean (SE). BMI, Body mass index, values are within the normal weight range for three profiles.

### Executive functions

For FT accuracy, preadolescents in Profile 1 and Profile 2 performed better than those in Profile 3 (1 vs. 3: *χ*^2^ = 12.23, *P* < 0.001; 2 vs. 3: *χ*^2^ = 10.86, *P* = 0.001). No group difference was observed between Profile 1 and Profile 2 (*χ*^2^ = 0.12, *P* = 0.73). No group difference was observed in FT reaction time (all *P* > 0.05) for the three profiles. No group difference was observed in SBT accuracy and reaction time for the three profiles (all *P* > 0.05) (see Table 4).

**Table 4:** Mean of physical fitness across test sample (*N* = 120): executive function.

	Profile 1	Profile 2	Profile 3	Overall Wald *χ*^2^
	*N* = 28	*N* = 43	*N* = 49	
Flanker task				
Reaction time (ms)	691.17 (26.99)	668.24 (20.92)	707.64 (28.67)	*χ*^2^ = 1.16, *P* = 0.56
Accuracy (%)	98.33 (0.94)***	97.89 (0.81)**	89.33 (2.31)	χ^2^ = 12.44, *P* = 0.002
Sternberg task				
Reaction time (ms)	926.49 (39.14)	893.46 (37.38)	923.06 (42.96)	χ^2^ = 0.41, *P* = 0.81
Accuracy (%)	94.72 (1.06)	94.58 (1.69)	92.45 (1.52)	χ^2^ = 1.46, *P* = 0.48

Data are presented as mean (SE). Profile 1 = high BMI—poor VO_2max_—slow 20-m sprint; Profile 2 = low BMI-fair VO_2max_—fast 20-m sprint; Profile 3 = low BMI—poor VO_2max_—slow 20-m sprint **P < 0.01, ***P < 0.001 compared with Profile 3.

## Discussion

Compared with the widely used variable-centered approach, the current study is the first to adopt a person-centered approach to explore the relationship between nutritional status (BMI) and physical fitness (CRF [predicted VO_2max_] and speed-agility [20-m sprint]) profiles and EF in preadolescents. Three distant profiles were identified: Profile 1 (high BMI, poor VO_2max_, and slow 20-m sprint). Profile 2 (low BMI, fair VO_2max_, and fast 20-m sprint) and Profile 3 (low BMI, poor VO_2max_, and slow 20-m sprint). Referring to the WHO guidelines, the nutritional status among the three profiles belongs to normal weight. Preadolescents in profiles 1 and 2 performed better on FT accuracy than those in profile 3. Results suggest that (1) among preadolescents with poor CRF and low speed-agility ability, those with superior nutritional status showed better inhibitory control performance than their peers with poor nutritional status; (2) among preadolescents with poor nutritional status, those with higher CRF and speed-agility showed better inhibitory control performance compared to preadolescents with worse physical fitness; (3) no association between profiles and working memory was observed.

The findings that preadolescents with poor CRF and low speed-agility showed better EF performance when they had higher BMI (superior nutritional status) appear to be at odds with the mainstream of the literature using the variable-centered approach. For example, a review study that pooled nine cross-sectional studies of the relationship between obesity and cognition in children and adolescents found that eight of the nine studies showed significantly worse EF indices in obese individuals than normal-weight individuals (37). Using a linear regression model, increasing BMI was associated with lower EF (5), and overweight or obese adolescents showed poorer inhibitory control than their normal-weight peers (4). From a neurophysiological perspective, high BMI is significantly related to the low cortical thickness of eighteen cortical regions, particularly the prefrontal cortex in preadolescents (38). In addition, BMI in obesity was found to be associated with decreased frontal and limbic gray matter volume (39). Both the reduced cortical thickness and volume of brain regions suggest the possibility of poorer EF performance.

However, this linear relationship does not always exist. According to Raine et al. (2018), visceral adipose tissue (VAT) was positively associated with EF in normal weight children (aged 8–10 years), but high VAT was associated with poor EF performance in children with obesity (40). The results suggest that VAT is selectively and negatively related to cognition in children with obesity, and thus the relationship between BMI and EF should be reconsidered based on children's BMI levels (i.e., obesity or not). In our study, preadolescents in profile 1 had, on average, a BMI in the upper range of normal urban Chinese weight but did not reach overweight (Cut-off, BMI = 21.61 kg/m^2^) (23, 25), indicating a superior nutritional status in normal weight. For this range, better nutritional status was found to have a positive effect on EF (40), which may explain the finding that profile 1 performed better than profiles 2 and 3. However, this finding is preliminary, and it is recommended that further RCT studies be designed to validate this finding.

Another plausible explanation is that BMI not only represents nutritional status but also predicts the developmental trajectories of children and adolescents (24). The better performance in profile 1 could be due to the fact that preadolescents developed earlier in this profile than in the other profiles, as there is a shift in the relationship between developmental domains as a function of age. Specifically, among Chinese children and adolescents, especially those in Hong Kong, BMI for girls increased from aged 9 to 13 and became stable at age 14 and 15; whereas BMI for boys increased from aged 9 to 11, became stable between aged 11 and 14 (from 17.4 to 20.0 kg/m^2^) (41). Participants in our sample were in a fast-developing phase, and the average BMI in profile 1 was slightly higher than the stable phase value mentioned above (i.e., >20.0 kg/m^2^), which may indicate that preadolescents in profile 1 were faster developed than preadolescents in the other profiles (e.g., 15.99 kg/m^2^ in profile 2 and 15.63 kg/m^2^ in profile 3). According to a previous study, preadolescents in older group (11–12 yrs) performed better in inhibition control (Flanker task) and working memory (2-back task) than younger group (9–10 yrs), and regression analysis showed that age was generally associated with better performances of executive function (42). Therefore, it is plausible that preadolescents in profile 1 developed faster than those in other profiles, leading to better cognition performance.

Consistent with previous variable-centered studies that physical fitness benefits EF in preadolescents, our second finding indicated that among preadolescents with poor nutritional status, those with higher CRF and speed-agility showed better inhibitory performance compared to preadolescents with worse physical fitness (43). The combination of CRF and speed-agility appears to promote EF together. According to a review, CRF was not associated with EF when speed-agility was controlled (44). An empirical study found that children with better results in CRF (predicted VO_2max_) and speed-agility (4 × 10 m shuttle run test) scored better in all cognitive dimensions, even after controlling for BMI (15). Nevertheless, CRF and speed-agility were also reported to be individually associated with EF. For example, after correcting for age, gender and BMI, Ludyga et al. (2019) observed that motor ability was associated with conflict score on the Flanker task in preadolescents (45). Drozdowska et al. (2021) observed a weak but significant positive correlation between speed (20-m sprint) and FT reaction time performance in a German study of 211 preadolescents (46). CRF (predicted VO_2max_) is generally reported to be associated with EF performance in Chinese preadolescents (42, 47). The possible mechanism is that participants who exhibited better physical fitness had better cerebral oxygenation (48, 49), and cognitive processing critically depends on adequate blood flow to meet the energy and oxygen demands of the brain's cortical tissue (50). According to the standard provided by a large Chinese sample study (51), preadolescents in profile 2 have a fair CRF with good speed-agility, and those in profile 3 have a poor CRF with worse speed-agility. Therefore, with poor nutritional status preadolescents, improving EF by promoting physical fitness (i.e., CRF and speed-agility) seems feasible. Preadolescents should engage in moderate to vigorous physical activity, which correlates positively with CRF and speed-agility and is recommended by the World Health Organization (52, 53).

No association was found between nutritional status, physical fitness profiles and working memory. This is consistent with a review study of differences in cognition between overweight and normal weight children, which found that only one of four individual studies reported the correlation between BMI and working memory (4). However, the correlation between the 20-m sprint and working memory is controversial. In a study using two tasks to measure working memory, the speed of the 20-m sprint was weakly but significantly related to the score of the Corsi-block tapping task, but not to the 2-back task (46). This discrepancy may be explained by a measurement bias in working memory that is common in neurobehavioral experiments. Regarding CRF, consistent with a UK study using the same cognitive battery with the same task as the current study (i.e., SBT, working memory), CRF (predicted VO_2max_) is not associated with the “three- and five- items” for both reaction time and accuracy (54). However, most recent studies assume that a higher CRF is correlated with better working memory. For example, Zhan et al. (2020) found that CRF level (predicted VO_2max_) was positively associated with response accuracy in the 2-backward task (42). A similar result was also observed after controlling for demographic characteristics (e.g., age, gender, grade) in preadolescents (55, 56). An intervention study found that increased CRF was associated with improved performance on the SBT in preadolescents (57). The mixed results indicate the need for further investigation, with the present study adding to the literature by using a person-centered (latent profile analysis) approach for the first time, compared to the more commonly used variable-centered approach.

There are a few limitations of our study. First, this study is cross-sectional and causality of the observed associations cannot be determined. Future studies should consider a rigorous study design such as RCT to better understand the associations and validate the results of our study. Second, we used single measurements for all variables. Future studies might consider using multiple measurements for the key variables, such as adding the Stroop task to measure inhibition, the Corsi-block tapping task and the 2-back task to measure working memory, and adding visceral adipose tissue as an indicator of the nutritional status.

## Conclusion

Compared with the commonly used variable-centered approach, this study, which took a person-centered approach, provided addictive information on the association between nutritional status, physical fitness profiles and EF in preadolescents. Findings suggest that for normal weight preadolescents, promoting EF by improving nutritional status may be effective for those with poor physical fitness. For preadolescents with poor nutritional status, improving CRF and speed-agility may be beneficial for EF.

## Data Availability

The raw data supporting the conclusions of this article will be made available by the authors, without undue reservation.
