# Corrigendum: Association between nutritional status, physical fitness and executive functions in preadolescents: a person-centered approach

**DOI:** 10.3389/fped.2024.1362657

**Published:** 2024-02-14

**Authors:** Yuxin Zhu, Fenghua Sun, Sisi Tao, Simon B. Cooper, Tian-Yu Gao

**Affiliations:** ^1^ Syns Institute of Educational Research, Hong Kong, Hong Kong SAR, China; ^2^ Department of Health and Physical Education, The Education University of Hong Kong, Hong Kong, Hong Kong SAR, China; ^3^ Centre for Information Technology in Education, Faculty of Education, The University of Hong Kong, Hong Kong, Hong Kong SAR, China; ^4^ Exercise/Health Research Group, Sport, Health and Performance Enhancement (SHAPE) Research Centre, Department of Sport Science, Nottingham Trent University, Nottingham, United Kingdom; ^5^ School of Physical Education, Jinan University, Guangzhou, China

**Keywords:** body mass index, exercise, latent profile analysis, inhibition, working memory, attention, cognition

A Corrigendum on Association between nutritional status, physical fitness and executive functions in preadolescents: a person-centered approach By Zhu Y, Sun F, Tao S, Cooper SB and Gao T-Y (2022). Front. Pediatr. 10:966510. doi: 10.3389/fped.2022.966510

In the published article there was an error, affiliations 6 “Institute for Intelligent Sport and Digital Health, Jinan University, Guangzhou, China” should be removed.

Furthermore, affiliation “School of Physical Education, Jinan University, Guangzhou, China” should be removed from author Yuxin Zhu.

The authors apologize for this error and state that this does not change the scientific conclusions of the article in any way. The original article has been updated.

